# *LRRK2* and *GBA1* variant carriers have higher urinary bis(monacylglycerol) phosphate concentrations in PPMI cohorts

**DOI:** 10.1038/s41531-023-00468-2

**Published:** 2023-02-28

**Authors:** Kalpana M. Merchant, Tanya Simuni, Janel Fedler, Chelsea Caspell-Garcia, Michael Brumm, Kelly N. H. Nudelman, Elizabeth Tengstrandt, Frank Hsieh, Roy N. Alcalay, Christopher Coffey, Lana Chahine, Tatiana Foroud, Andrew Singleton, Daniel Weintraub, Samantha Hutten, Todd Sherer, Brit Mollenhauer, Andrew Siderowf, Caroline Tanner, Ken Marek, the Parkinson’s Progression Markers Initiative

**Affiliations:** 1 Northwestern University Feinberg School of Medicine, Chicago, IL USA; 2 The University of Iowa, Iowa City, IA USA; 3 Indiana University School of Medicine, Indianapolis, IN USA; 4 Nextcea, Inc, Woburn, MA USA; 5 Columbia University, New York, NY USA; 6 University of Pittsburg, Pittsburgh, PA USA; 7 Indiana University, Indianapolis, IN USA; 8 National Institute on Aging, NIH, Bethesda, MD USA; 9 The University of Pennsylvania, Philadelphia, PA USA; 10 The Michael J Fox Foundation for Parkinson’s Research, New York, NY USA; 11 University Medical Center Goettingen, Parcacelsus Kliniken Germany, Göttingen, Germany; 12 University of California San Francisco, San Francisco, CA USA; 13 Institute for Neurodegenerative Disorders, New Haven, CT USA

**Keywords:** Neurological disorders, Parkinson's disease

## Abstract

We quantified concentrations of three isoforms of the endolysosomal lipid, bis(monoacylglycerol) phosphate (BMP) in the urine of deeply phenotyped cohorts in the Parkinson’s Progression Markers Initiative: *LRRK2* G2019S PD (*N* = 134) and non-manifesting carriers (NMC) (G2019S+ NMC; *N* = 182), *LRRK2* R1441G PD (*N* = 15) and R1441G+ NMC (*N* = 15), *GBA1* N409S PD (*N* = 76) and N409S+ NMC (*N* = 178), sporadic PD (sPD, *N* = 379) and healthy controls (HC) (*N* = 190). The effects of each mutation and disease status were analyzed using nonparametric methods. Longitudinal changes in BMP levels were analyzed using linear mixed models. At baseline, all *LRRK2* carriers had 3–7× higher BMP levels compared to HC, irrespective of the disease status. *GBA1* N409S carriers also showed significant, albeit smaller, elevation (~30–40%) in BMP levels compared to HC. In *LRRK2* G2019S PD, urinary BMP levels remained stable over two years. Furthermore, baseline BMP levels did not predict disease progression as measured by striatal DaT imaging, MDS-UPDRS III Off, or MoCA in any of the cohorts. These data support the utility of BMP as a target modulation biomarker in therapeutic trials of genetic and sPD but not as a prognostic or disease progression biomarker.

## Introduction

Rare missense mutations in the gene encoding a multidomain protein, leucine-rich repeat kinase 2 (LRRK2), are a major cause of autosomal dominant Parkinson’s disease (PD)^1,2^, whereas common variants in the *LRRK2* gene are associated with sporadic PD^3,4^. Variants in *GBA1*, the gene encoding the lysosomal hydrolase, glucocerebrosidase (GCase), represent the most common genetic risk factors for PD and related synucleinopathies^5^. The precise mechanisms by which *LRRK2* or *GBA1* mutations cause PD is not entirely clear. However, recent cell biological studies have begun to implicate endolysosomal trafficking and lysosomal dysfunction as key pathogenic mechanisms associated with *LRRK2*- and *GBA1*-associated PD^6–8^. In aggregate, these studies have spurred a robust pipeline of therapeutics targeting LRRK2 and GCase^9,10^. We have recently shown that LRRK2 kinase negatively regulates the activity of lysosomal GCase in human induced pluripotent cells^11^, further demonstrating a convergence between LRRK2 and GCase pathways^12^. It has been suggested that LRRK2-and GCase-targeted therapies may benefit sporadic PD (sPD) also on the basis of an increase in LRRK2 kinase activity^13^ and a deficiency in GCase^14^ in brains of sPD cases, though the former data have not been replicated broadly^6,15^. The heterogeneity of sPD and low penetrance of *LRRK2*^16^ and *GBA1*^5^ variants for PD necessitate a patient enrichment strategy to support clinical development of therapies. Thus, biomarkers that could detect endolysosomal dysfunction may inform patient enrichment strategies for LRRK2- and GCase-targeted therapeutics. Additionally, such biomarkers could also constitute pharmacodynamic markers to demonstrate relevant target modulation at clinically used doses.

Several lines of evidence indicate that bis(monoacylglycerol)phosphate (BMP), previously called LBPA (lysophosphosphatidic acid), is an atypical phospholipid that regulates and is affected by endolysosomal functions^17–19^. We recently examined 11 isoforms of BMP in the urine from two independent LRRK2 cohorts and observed an elevation in all BMP isoforms assayed in *LRRK2* mutation carriers compared to non-carriers^20^. A correlational analysis indicated that total di-18:1-BMP and total di-22:6-BMP most strongly discriminated the *LRRK2* mutation carriers from non-carriers. Furthermore, levels of di-22:6-BMP and its 2,2′ isoform negatively correlated with MoCA scores in the carriers. However, this study had two major shortcomings. One, it was performed in smaller cross-sectional cohorts that lacked deep phenotyping data which precluded our ability to assess correlations between BMP levels and clinical progression. Second, some participants in the *LRRK2* cohort also carried *GBA1* pathogenic variants, which may have confounded outcomes and data interpretation. Thus, further investigation of urinary BMP as a biomarker that could facilitate PD therapeutic development is needed. On the basis of our previous study, we focused the current study on the assessment of total di-18:1-BMP, total di-22:6-BMP, and 2,2′-di-22:6-BMP.

The objectives of the present study were to utilize the deeply phenotyped and large longitudinal observational cohorts from the Parkinson’s Progression Markers Initiative (PPMI) to extend our previous studies^20^ and assess: (a) effects of *LRRK2* and *GBA1* pathogenic variants on baseline urinary BMP levels in PD manifesting and non-manifesting carriers (NMC), (b) longitudinal changes in BMP levels in *LRRK2* carriers, and (c) whether baseline BMP levels predict disease progression in *LRRK2*, *GBA1* or sPD cohorts.

## Results

### Baseline BMP concentrations are higher in *LRRK2* cohorts compared to sPD and HC

**Fig. 1:**
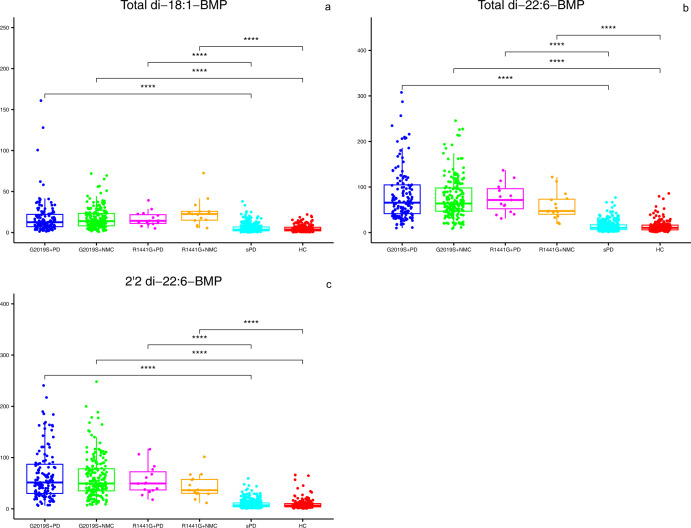
Levels of three BMP isoforms in *LRRK2* G2019S+ and R1441G+ individuals compared to HC and sPD. **a**–**c** Plots show baseline concentrations of each specific BMP isoform in the six groups indicated on the *X* -axis. Box-and-whisker plots with scatter plots superimposed are shown with each filled circle representing observed data. The bottom and top edges of the box correspond to the 75th and 25th percentiles, respectively. The horizontal line within the box indicates the median. The whiskers indicate the range of values within 1.5 times the interquartile range. A Rank-based linear model with adjustment for age and sex was used for pairwise comparisons. The significance level for pairwise comparisons is *p* value <0.0125 (after Bonferroni correction). ****p value <0.0001.

Demographics, baseline clinical characteristics and mean striatal specific binding ratios (SBR) computed from the dopamine transporter (DaT) SPECT imaging data (DaTscan) for PD manifesting and NMC of *LRRK2* G2019S+ and R1441G+ were compared to sPD and HC groups (Table 1a, b, respectively). Neither G2019S+ nor R1441G+ carriers with PD or NMC differed significantly in age from sPD or HC, respectively. Consistent with PPMI’s enrollment strategy, disease duration at baseline was significantly longer for both G2019S+ and R1441G+ PD manifesting groups compared to the sPD group. Consequently, the *LRRK2* G2019S+ PD group showed greater deficits in mean striatal DaT binding and MoCA than sPD. Similarly, the R1441G+ PD group showed greater deficits in MoCA and UPDRS III Off than the sPD cohort. The sex distribution was also different between sPD and *LRRK2* genetic cohorts. Baseline BMP concentrations for each LRRK2 genotype were compared across the four groups after adjusting for age and sex. Consistent with our previous report^20^, concentrations of all three BMP isoforms were significantly higher (3–7-fold) in *LRRK2* G2019S+ carriers compared to HC and sPD, but the levels did not differ between the PD manifesting and NMC groups (Table 1a and Fig. 1). This effect of *LRRK2* genotype on BMP elevation, irrespective of disease status, was also seen in R1441G+ carriers (Table 1b and Fig. 1). A comparison of baseline BMP levels in G2019S+ and R1441G+ sub-cohorts showed statistically significant overall effect and higher levels in R1441 G+ NMC sub-cohort compared to G2019S+ NMC (Supplemental Table 1). Unlike the genetic cohort, the sPD group did not show an elevation in any BMP isoform when compared to HC (Table 1a).

**Table 1:** Baseline demographics, clinical characteristics, mean striatal DaT SBR and BMP concentrations (adjusted for age and sex) in LRRK2, sPD and HC cohorts.

a Comparison of LRRK2 G2019S sub-cohorts to sPD and HC								
Cohort	1	2	3	4				
	G2019S + PD *n* = 134	G2019S + NMC *n* = 182	sPD *n* = 379	HC *n* = 190	*P* Value 1 vs 2	*P* Value 1 vs 3	*P* Value 2 vs 4	*P* Value 3 vs 4
Age (years), mean (SD)^a^	63.26 (9.20)	62.33 (7.72)	61.94 (9.66)	60.67 (11.16)				
Age at onset (years), mean (SD)	58.05 (9.99)		59.94 (9.93)		N/A	0.0609	N/A	N/A
Mean Striatal DaT SBR, mean (SD) missing	1.29 (0.38)	2.52 (0.52)	1.39 (0.39)	2.57 (0.56)	**<0.0001**	**0.0093**	0.4164	**<0.0001**
	15	9	4	3				
Disease duration (months), median (range)	28.55		4.2		N/A	**<0.0001**^α^	N/A	N/A
	(0.97, 104.43)		(0.40, 35.83)					
Female Sex, n (%)	65 (48.51%)	104 (57.14%)	129 (34.04%)	67 (35.26%)	0.1283	**0.0030**	**<0.0001**	0.7716
MDS-UPDRS III Off, mean (SD) missing	22.46 (11.45)	3.14 (4.11)	20.89 (8.79)	1.19 (2.15)	**<0.0001**	0.2097	**<0.0001**	**<0.0001**
	36	0	0	2				
MoCA, median (range) missing	27	27	28	28	0.2689^α^	**0.0010**^α^	**<0.0001**^α^	**<0.0001**^α^
	(13.00, 30.00)	(18.00, 30.00)	(17.00, 30.00)	(26.00, 30.00)				
	2	1	0	0				
Total di-18:1-BMP, median (range)	12.60	13.88	3.53	3.77	0.2246	**<0.0001**	**<0.0001**	0.8823
	(1.29, 160.95)	(0.90, 71.97)	(0.21, 38.02)	(0.39, 22.03)				
Total di-22:6-BMP, median (range)	65.63	63.79	10.61	10.42	0.8082	**<0.0001**	**<0.0001**	0.5976
	(9.85, 307.69)	(8.65, 245.60)	(1.18, 76.76)	(1.25, 85.89)				
2,2’-di-22:6-BMP, median (range)	51.39	49.38	6.83	6.37	0.9820	**<0.0001**	**<0.0001**	0.6946
	(6.22, 240.68)	(6.99, 248.17)	(0.81, 59.27)	(0.60, 65.96)				

b Comparison of LRRK2 R1441G sub-cohorts to sPD, and HC							
Cohort	1	2	3	4			
	R1441G + PD *n* = 15	R1441G + NMC *n* = 15	sPD *n* = 379	HC *n* = 190	*P* Value 1 vs 2	*P* Value 1 vs 3	*P* Value 2 vs 4
Age (years), mean (SD)	64.51 (10.46)	56.82 (5.09)	61.94 (9.66)	60.67 (11.16)	0.0186	0.3650	0.0192
Age at onset (years), mean (SD)	60.70 (11.74)		59.94 (9.93)		N/A	0.8069	N/A
Mean Striatal DaT SBR, mean (SD) missing	1.37 (0.33)	2.64 (0.68)	1.39 (0.39)	2.57 (0.56)	**<0.0001**	0.7551	0.7039
	0	0	4	3			
Disease duration (months), median (range)	24.8		4.2		N/A	**<0.0001**^**α**^	N/A
	(4.10, 77.80)		(0.40, 35.83)				
Female Sex, *n* (%)	11 (73.33%)	10 (66.67%)	129 (34.04%)	67 (35.26%)	0.6903	**0.0018**	0.0156
MDS-UPDRS III Off, mean (SD) missing	15.86 (5.57)	2.29 (3.31)	20.89 (8.79)	1.19 (2.15)	**<0.0001**	**0.0053**	0.2421
	1	1	0	2			
MoCA, median (range) missing	25	28	28	28	0.0494^α^	**0.0011**^**α**^	0.0812^α^
	(18.00, 30.00)	(22.00, 30.00)	(17.00, 30.00)	(26.00, 30.00)			
	0	0	0	0			
Total di-18:1-BMP, median (range)	14.25	22.65	3.53	3.77	0.3133	**<0.0001**	**<0.0001**
	(5.04, 39.26)	(5.40, 72.51)	(0.21, 38.02)	(0.39, 22.03)			
Total di-22:6-BMP, median (range)	71.52	47.27	10.61	10.42	0.3584	**<0.0001**	**<0.0001**
	(30.90, 136.67)	(19.15, 121.85)	(1.18, 76.76)	(1.25, 85.89)			
2,2’-di-22:6-BMP, median (range)	49.43	36.29	6.83	6.37	0.2203	**<0.0001**	**<0.0001**
	(17.82, 116.30)	(11.50, 101.43)	(0.81, 59.27)	(0.60, 65.96)			

Pairwise comparisons that showed significance at *p* value <0.0125 (after Bonferroni correction applied due to multiple comparison) are shown in bolded font.

*PD* Parkinson’s disease, *NMC* non-manifesting carriers, *sPD* sporadic PD, *HC* healthy control, *SD* standard deviation, *DaT* dopamine transporter, *SBR* specific binding ratio, *MDS-UPDRS* movement disorders society-unified Parkinson’s disease rating scale, *MoCA* Montreal cognitive assessment, BMP bis(monoacylglycerol)phosphate.

^α^Computed using Mann–Whitney *U*-test.

aThe overall *F*-test was non-significant thus pairwise comparisons were not computed.

### Longitudinal Changes in BMP levels in *LRRK2* mutation carriers

Given the robust increases in baseline BMP levels associated with *LRRK2* genotypes, we examined whether BMP isoforms change over time in *LRRK2* G2019S+ or R1441G+ individuals manifesting PD and *LRRK2* NMCs. Table 2a, b show annualized change in BMP levels and an assessment of whether disease status affects the longitudinal change in *LRRK2* G2019S+ and R1441G+ carriers (note the log of the BMP level was modeled due to the skewed distribution of the data). In the G2019S+ PD group, di-18:1-BMP increased longitudinally whereas the R1441G+ individuals showed quantitatively greater changes in all three BMP isoforms in the NMC group. However, there was no statistically significant difference in longitudinal BMP changes in the PD manifesting versus NMC groups for either *LRRK2* genotype. Supplemental Table 2 shows the raw concentrations of each BMP isoform at baseline, year 1 and year 2 by disease status for the two *LRRK2* genotypes. These data can be useful to facilitate power calculations for future studies.

**Table 2:** Estimated annualized change in log BMP concentrations in LRRK2 cohorts.

BMP isoform	Disease status	Estimated log change over 12 months (95% CI)	*P* value
a LRRK2 G2019S+ sub-cohorts.			
Total di-18:1-BMP	PD	0.0677 (0.0020, 0.1335)	**0.0435**
	NMC	0.0529 (−0.0039, 0.1096)	0.0677
	PD vs NMC	0.0149 (−0.0720, 0.1017)	0.7363
Total di-22:6-BMP	PD	0.0144 (−0.0379, 0.0668)	0.5882
	NMC	0.0312 (−0.0139, 0.0764)	0.1748
	PD vs NMC	−0.0168 (−0.0860, 0.0524)	0.6332
2,2’ di-22:6-BMP	PD	0.0127 (−0.0471, 0.0725)	0.6768
	NMC	0.0203 (−0.0313, 0.0718)	0.4404
	PD vs NMC	−0.0076 (−0.0865, 0.0714)	0.8505

b LRRK2 R1441G+ sub-cohorts.			
Total di-18:1-BMP	PD	0.1046 (−0.0763, 0.2855)	0.2503
	NMC	0.1901 (0.0074, 0.3727)	**0.0417**
	PD vs NMC	−0.0854 (−0.3427, 0.1718)	0.5073
Total di-22:6-BMP	PD	0.0903 (−0.0235, 0.2041)	0.1172
	NMC	0.1344 (0.0189, 0.2498)	**0.0235**
	PD vs NMC	−0.0441 (−0.2063, 0.1181)	0.5869
2,2’ di-22:6-BMP	PD	0.1172 (−0.0159, 0.2502)	0.0829
	NMC	0.1433 (0.0085, 0.2781)	**0.0377**
	PD vs NMC	−0.0262 (−0.2157, 0.1633)	0.7824

Linear Mixed Models (LMM) were used to estimate the annualized change in log BMP concentrations with fixed effects of disease status and time and interaction between them after adjusting for age and sex. Significant *P* values are shown in bolded font.

*PD* Parkinson’s disease, *NMC* non-manifesting carriers, *BMP* bis(monoacylglycerol)phosphate.

### Baseline BMP concentrations are higher in the *GBA1* N370S+ cohort, but not in other *GBA1* genotypes compared to sPD and HC

**Fig. 2:**
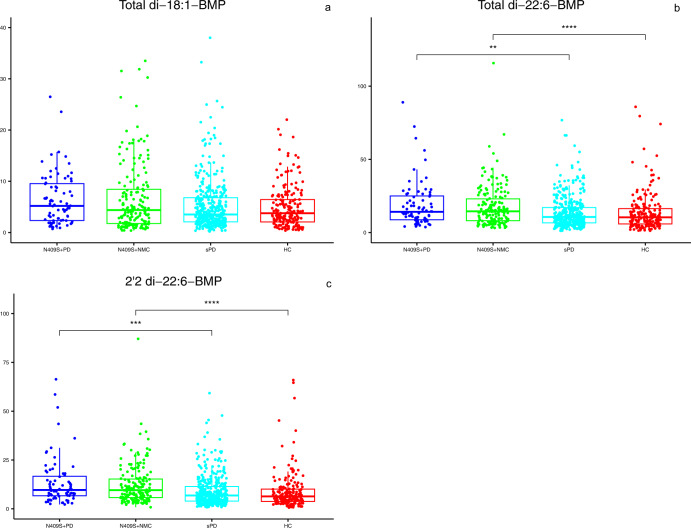
Levels of three BMP isoforms in *GBA1* N409S carriers compared to HC and sPD. **a**–**c** Plots show baseline concentrations of each specific BMP isoform measured in the 4 groups indicated on the *X*-axis. Box-and-whisker plots with scatter plots superimposed are shown with filled circles representing observed data. The bottom and top edges of the box correspond to the 75th and 25th percentiles, respectively. The horizontal line within the box indicates the median. The whiskers indicate the range of values within 1.5 times the interquartile range. Mann–Whitney *U*-test was used for pairwise comparisons. The significance level for pairwise comparisons is *p* value <0.0125 (after Bonferroni correction). ****p value <0.0001, ***p value <0.0005, and **p value <0.005.

Given the previously reported convergence in cellular processes affected by LRRK2 and GCase, we assessed whether risk variants of *GBA1* affect urinary BMP levels. Table 3 shows demographics and baseline characteristics for *GBA1* N409S+ PD (*N* = 76) and NMC (*N* = 178) compared with the sPD (*N* = 379) and HC (*N* = 190) groups. There was no overall difference in age between the *GBA1* N409S+ groups, HC, or sPD. As with the *LRRK2* G2019S+ /R1441G+ PD groups, the *GBA1* N409S+ PD had significantly longer disease duration and associated higher MDS-UPDRS III Off and lower MoCA scores when compared to sPD. However, unlike the lower mean striatal DaT SBR in the G2019S+ PD group when compared to sPD (Table 1a), the *GBA1* N409S+ PD group did not differ in striatal SBR from sPD. Interestingly, total di-22:6-BMP and 2,2′ di-22:6-BMP isoforms were higher in *GBA1* N409S+ individuals (PD and NMC) but not total di-18:1-BMP. However, the levels of di-22:6-BMP isoforms did not differ between those with and without PD (Table 3 and Fig. 2), once again indicating that BMP is a trait marker even in the *GBA1* carriers. It is noteworthy that the magnitude of the increase in BMP concentration in N409S+ was ~40%, much smaller than the 3–7X elevation seen with the *LRRK2* genotypes.

**Table 3:** Baseline demographics, clinical characteristics, mean striatal DaT SBR and BMP concentrations (adjusted for age and sex) in *GBA1* N409S + PD, N409S + NMC, sPD, and HC.

	1	2	3	4			
	N409S+ PD *n* = 76	N409S+ NMC *n* = 178	sPD *n* = 379	HC *n* = 190	*P* value 1 vs 2	*P* value 1 vs 3	*P* value 2 vs 4
Age (years), mean (SD)^a^	62.79 (10.22)	62.15 (6.71)	61.94 (9.66)	60.67 (11.16)			
Age at onset (years), mean (SD)	58.52 (10.88)		59.94 (9.93)		N/A	0.2945	N/A
Mean Striatal DaT SBR, mean (SD) missing	1.28 (0.52)	2.79 (0.57)	1.39 (0.39)	2.57 (0.56)	**<0.0001**	0.1003	0.7039
	13	5	4	3			
Disease duration (months), median (range)	31.72		4.2		N/A	**<0.0001**^α^	N/A
	(0.30, 85.23)		(0.40, 35.83)				
Female Sex, n (%)	33 (43.42%)	106 (59.55%)	129 (34.04%)	67 (35.26%)	0.0180	0.1189	**<0.0001**
MDS-UPDRS III Off, mean (SD) missing	26.87 (11.11)	2.60 (3.90)	20.89 (8.79)	1.19 (2.15)	**<0.0001**	**0.0001**	**<0.0001**
	15	1	0	2			
MoCA, median (range) missing	27	27	28	28	0.2107^α^	**0.0056**^α^	**<0.0001**^α^
	(15.00, 30.00)	(16.00, 30.00)	(17.00, 30.00)	(26.00, 30.00)			
	0	0	0	0			
Total di-18:1-BMP, median (range)^b^	5.20	4.38	3.53	3.77			
	(0.70, 26.49)	(0.38, 33.51)	(0.21, 38.02)	(0.39, 22.03)			
Total di-22:6-BMP, median (range)	14.14	14.51	10.61	10.42	0.9184	**0.0014**	**<0.0001**
	(3.74, 88.97)	(3.16, 115.74)	(1.18, 76.76)	(1.25, 85.89)			
2,2’-di-22:6-BMP, median (range)	9.65	9.54	6.83	6.37	0.9248	**0.0001**	**<0.0001**
	(2.22, 66.35)	(0.81, 87.08)	(0.81, 59.27)	(0.60, 65.96)			

Pairwise comparisons that showed significance at *p* value <0.0125 (after Bonferroni correction applied due to multiple comparisons) are shown in bolded font.

*PD* Parkinson’s disease, *NMC* non-manifesting carriers, *sPD* sporadic PD, *HC* healthy control, *SD* standard deviation, *DaT* dopamine transporter, *SBR* specific binding ratio, *MDS-UPDRS* movement disorders society-unified Parkinson’s disease rating scale, MoCA Montreal cognitive assessment, BMP bis(monoacylglycerol)phosphate.

^α^Computed using Mann–Whitney *U*-test.

^a^The overall *F*-test was non-significant thus pairwise comparisons were not computed.

^b^The overall test was non-significant thus pairwise comparisons were not computed.

We also examined baseline BMP levels in three additional *GBA1* sub-cohorts, though each had a small number of individuals: those associated with severe Gaucher disease, L483P (*N* = 8 with PD and *N* = 3 NMC), and a group of rare variants combined (IVS2+ 1 G > A, L29Afs18, T408M+ R159W, R502C; *N* = 6 with PD and *N* = 4 NMC) as well as the PD-specific common risk variant, E365K (*N* = 19 with PD and *N* = 5 NMC). Surprisingly, there was no overall difference in baseline BMP levels in these *GBA1* sub-cohorts when compared to sPD, and HC (Supplemental Table 3a–c).

A small number of *LRRK2* G2019S carriers also carried the *GBA1* N409S variant (*N* = 5 with PD and *N* = 15 NMC). Although the small numbers do not allow definitive conclusions, BMP levels in these individuals had a similar pattern to those seen in *LRRK2* G2019S+ subjects, i.e., BMP levels were elevated in the compound mutation carriers, but did not differ between those with and without PD (Supplemental Table 4). When comparing BMP levels in the *LRRK2* G2019S+ with those with both *LRRK2* G2019S+ and *GBA1* N409S+, there was no overall statistical difference in concentrations of any BMP isoform indicating that there is no interaction between these genotypes in the sub-cohorts studied.

### Baseline BMP levels do not predict a decline in striatal DaT and clinical outcomes over 5 years

Our final objective was to examine whether baseline BMP isoforms could predict a longitudinal decline in striatal DaT, MDS-UPDRS III Off, or MoCA in the two largest genetic groups, *LRRK2* G2019S+, and *GBA1* N409S+ as well as the sPD and HC group (note that the HC group is excluded from the modeling of the DaT data since this group did not undergo DaT SPECT following baseline visit). Overall, we did not observe a relationship between any isoform of BMP at baseline and the change in striatal DaT SBR, MDS- UPDRS III Off, or MoCA in either genetic cohort or sPD and HC group (Table 4a–c). Since *LRRK2* R1441G is thought to be more pathogenic than *LRRK2* G2019S, we explored whether baseline BMP would predict clinical or radiographical decline and failed to see an association (Table 4a–c). The summary statistics for each outcome are shown in Supplementary Table 5a–c.

**Table 4:** Examination of an association between baseline BMP concentrations and longitudinal changes in DaT, MoCA or MDS-UPDRS III off.

Variant	Effect	Estimate	95% CI		*P* value
a Baseline BMP concentrations are not associated with longitudinal change in mean striatal DaT SBR					
G2019S+	Total di-18:1-BMP*time	0.00001	−0.0001	0.0001	0.7806
	Total di-22:6-BMP*time	0.00001	−0.00002	0.00003	0.6726
	2,2’ di-22:6-BMP*time	0.000002	−0.00003	0.00003	0.8718
R1441G+	Total di-18:1-BMP*time	0.0003	−0.000002	0.0007	0.0512
	Total di-22:6-BMP*time	−0.0001	−0.0002	0.0001	0.3025
	2,2’ di-22:6-BMP*time	−0.0001	−0.0003	0.0001	0.3199
N409S+	Total di-18:1-BMP*time	0.00005	−0.0003	0.0004	0.7988
	Total di-22:6-BMP*time	0.00008	−0.00008	0.0002	0.3231
	2,2’ di-22:6-BMP*time	0.0001	−0.0001	0.0003	0.3074
sPD*	Total di-18:1-BMP*time	0.00004	−0.00008	0.0002	0.4884
	Total di-22:6-BMP*time	0.000003	−0.00005	0.00005	0.9157
	2,2’ di-22:6-BMP*time	−0.000004	−0.00007	0.00007	0.9208

b Baseline BMP concentrations are not associated with longitudinal change in MoCA.					
G2019S+	Total di-18:1-BMP*time	−0.0001	−0.0005	0.0002	0.4543
	Total di-22:6-BMP*time	−0.00004	−0.0002	0.0001	0.5437
	2,2’ di-22:6-BMP*time	−0.00002	−0.0002	0.0001	0.8290
R1441G+	Total di-18:1-BMP*time	0.0022	0.0001	0.0044	0.0418
	Total di-22:6-BMP*time	−0.0003	−0.0010	0.0004	0.3532
	2,2’ di-22:6-BMP*time	−0.0005	−0.0013	0.0003	0.2060
N409S+	Total di-18:1-BMP*time	0.0006	−0.0011	0.0023	0.4694
	Total di-22:6-BMP*time	0.0002	−0.0006	0.0009	0.6190
	2,2’ di-22:6-BMP*time	0.0003	−0.0007	0.0013	0.5618
sPD and HC	Total di-18:1-BMP*time	−0.0011	−0.0019	−0.0002	0.0108
	Total di-22:6-BMP*time	−0.0002	−0.0005	0.0001	0.1796
	2,2’ di-22:6-BMP*time	−0.0003	−0.0007	0.0001	0.1554

c Baseline BMP concentrations are not associated with longitudinal change in MDS-UPDRS III Off.					
G2019S+	Total di-18:1-BMP*time	0.0002	−0.0019	0.0024	0.8273
	Total di-22:6-BMP*time	−0.0004	−0.0012	0.0004	0.3223
	2,2’ di-22:6-BMP*time	−0.0005	−0.0014	0.0004	0.3022
R1441G+	Total di-18:1-BMP*time	0.0077	−0.0021	0.0176	0.1186
	Total di-22:6-BMP*time	−0.0037	−0.0070	−0.0004	0.0314
	2,2’ di-22:6-BMP*time	−0.0039	−0.0079	0.0002	0.0591
N409S+	Total di-18:1-BMP*time	−0.0003	−0.0105	0.0099	0.9495
	Total di-22:6-BMP*time	0.0008	−0.0039	0.0055	0.7496
	2,2’ di-22:6-BMP*time	0.0002	−0.0060	0.0064	0.9457
sPD and HC	Total di-18:1-BMP*time	0.0009	−0.0027	0.0046	0.6230
	Total di-22:6-BMP*time	0.0002	−0.0011	0.0016	0.7492
	2,2’ di-22:6-BMP*time	0.0003	−0.0015	0.0021	0.7462

## Discussion

Here we present the largest dataset of analysis of BMP isoforms in individuals with the most common pathogenic/risk variants in *LRRK2* (G2019S and R1441G) and *GBA1* (N409S) as well as other PD-associated *GBA1* variants and compared them to sPD and HC. In the *LRRK2* carriers, our results indicate that: (i) baseline BMP levels are elevated in both G2019S+ and R1441G+ individuals, (ii) the elevation in baseline BMP levels did not differ between variant carriers+ PD and NMC, (iii) baseline total di-18:1-BMP levels are higher in R1441G+ NMC when compared to G2019S+ NMC sub-cohort but not in PD manifesting groups for the two *LRRK2* variants, (iv) longitudinal changes in BMP levels over 2 years do not differ between PD manifesting and NMC *LRRK2* individuals, and (v) baseline BMP does not predict 5-year longitudinal changes in DaT, MDS-UPDRS III Off or MoCA. In the *GBA1* cohort, we report that: (i) baseline BMP levels are elevated, but only in the N409S+ carriers; although the magnitude of the increase is significantly smaller than that in the *LRRK2* cohort (ii) there is no difference in BMP levels between N409S+ PD and N409S+ NMC, and (iii) baseline BMP levels are not associated with longitudinal changes in DaT, MDS-UPDRS III OFF or MoCA. Finally, we also failed to see an association between baseline BMP levels and longitudinal changes in DaT, MDS-UPDRS III off, or MoCA in sPD.

BMP, first identified in 1967, is a negatively charged glycerophospholipid with an unusual structure and is localized almost exclusively to late endosomal and lysosomal membranes^19^. BMPs contain two fatty acid acyl chains, the composition of which is cell-type specific and contributes to the biochemical functions of the various BMP species. Here we examined levels of total di-18:1-BMP and di-22:6-BMP on the basis of findings from our previous study^20^ showing that these two analogs are the most abundant in the urine and also most significantly associated with the *LRRK2* G2019S genotype. In addition, we examined the 2,2′ isomer of di-22:6-BMP since it is the major isoform in the late endosomes that is more active at forming multivesicular bodies than other BMP isoforms^21^ and was associated with worse cognition as measured by MoCA^20^. Key roles of BMPs include lysosomal stability, hydrolase activity, vesicle formation, and endosomal trafficking^19^. Furthermore, BMP accumulates in several lysosomal storage disorders, including Niemann-Pick disease^22,23^, a lysosomal disorder caused by diminished acid sphingomyelinase levels. Interestingly, variants in the *SMPD1* gene encoding acid sphingomyelinase are also risk factors for PD^24^. BMP accumulation has also been reported in Gaucher’s disease^19^, as well as iatrogenic phospholipidosis^25,26^. Thus, BMP appears to be an effective marker of endolysosomal homeostatic functions that could be interrogated in PD cohorts and controls.

Here we observed 4–7× elevation in baseline BMP isoform levels in the *LRRK2* G2019S+ group compared to HC, which is consistent with our previous report^20^. Additionally, we observed a similar magnitude of increase in those with the *LRRK2* R1441G variant despite a smaller number of individuals in this sub-cohort of the PPMI study. However, in the NMC sub-cohorts for the two LRRK2 variants, urinary total di-18:1-BMP levels were significantly higher in the R1441G+ than in G2019S+ individuals. It is noteworthy that disease status did not affect BMP levels in either G2019S+ or R1441G+ individuals, indicating that urine BMP is a trait, but not a state, marker. In the G2019S+ carriers, urinary BMP isoforms remained stable over two years in both PD-manifesting and non-manifesting individuals. On the other hand, R1441G+ carriers showed a significant and somewhat greater (~15%) annualized increase in BMP concentration in the NMC group, although there was no statistical difference in the longitudinal change between those with and without PD. R1441G is a rarer but more penetrant variant of *LRRK2* than G2019S. Whether the higher baseline levels of total di-18:1-BMP or the apparent higher rate of increase in BMP levels in R1441G is due to its greater pathogenicity would require further studies with a larger sample size.

Another novel finding of the present study is that *GBA1* N409S was also associated with significantly higher levels of total di-22:6 and 2, 2′-di-22:6-BMP at baseline, albeit the magnitude of increase was only ~30–40% when compared to those without the variant. Although the levels of total di-18:1-BMP were numerically higher in the *GBA1* N409S carriers, they did not reach statistical significance. Whether the apparent isoform-specific changes in *LRRK2* vs. *GBA1* variant carriers reflect cell-type specific effects of these variants or a reflection of the relatively smaller magnitude of change associated with the N409S variant remains to be seen. Future cell biological studies of mechanisms associated with BMP regulation by LRRK2 and GCase activity could shed light on this issue. As with *LRRK2*, there was no difference between baseline BMP concentrations for N409S+ with PD as compared to NMCs, indicating again that urine BMP is a trait marker of the N409S genotype. *GBA1* N409S significantly reduces GCase activity and causes generally mild and non-neuronopathic Gaucher disease and a mild clinical phenotype in PD^27,28^. Hence, we also examined BMP concentrations in the urine from Gaucher disease-associated severe *GBA1* variant carriers. Unexpectedly, there was no elevation in baseline BMP levels in these PPMI participants. It is possible that the lack of association of the severe Gaucher disease *GBA1* variants to urinary BMP levels was due to the small numbers of individuals with L483P (*N* = 10) and the group of rare point/frame-shift mutations (*N* = 11). Another *GBA1* variant in the PPMI study is E365K (*N* = 24), a PD risk factor that does not cause Gaucher disease in homozygote carriers. We did not observe an elevation in BMP levels in E365K+ carriers either. Given the high prevalence of this variant in the United States population, it would be important to examine BMP levels in a larger cohort of E365K+ to ensure that the limited data available in PPMI was not a contributing factor. In fact, assessing BMP levels across *GBA1* variants in larger cohorts is critical to assess whether a correlation exists between GCase activity and BMP concentrations^28^.

In our previous paper^20^, we proposed that LRRK2-mediated phosphorylation of Rab substrates^8^ may increase BMP concentrations in biofluids by affecting the biogenesis, motility, or extracellular release of endolysosomal vesicles. Indeed, a recent paper indicates that inhibition of LRRK2 kinase activity reduces the release of BMP-containing vesicles from the kidney^29^. Thus the observed increase in urinary BMP levels in *LRRK2* G2019S+ and R1441G+ individuals is likely due to the higher kinase activity of these pathogenic variants. On the other hand, the *GBA1* N409S variant reduces GCase activity and thereby induces lysosomal stress, which may, in turn, affect lysosomal vesicular release *via* a mechanism distinct from LRRK2-mediated BMP release. Ongoing cell biological studies investigating the regulation of biosynthesis, metabolism, and secretion of BMP by LRRK2 and GCase would provide insights into the mechanisms associated the observed increases in urinary BMP levels reported here. In our previous unbiased proteomics study also, we observed minimal overlap in lysosomal proteins affected by LRRK2 G2019S and GBA1 N409S variants, suggesting that the two variants affect distinct cell biological pathways^30^. Regardless of the mechanism, our observation that PD-associated variants in *LRRK2* and *GBA1* increase urinary BMP levels further indicates that the LRRK2 and GCase pathways converge on endolysosomal dysfunction. Our previous cell biology studies in iPS-derived dopaminergic neurons have also shown a convergence between LRRK2 and GCase^11^. There were only twenty individuals with both G2019S+ and N409S+ mutations (5 with PD), precluding our ability to reach firm conclusions around the interaction between these genotypes in the regulation of urinary BMP. However, numerically, these double mutation carriers showed a similar pattern of BMP elevation as seen in *LRRK2* variant carriers without an overall statistical difference when compared to G2019S+ sub-cohort.

We examined BMP levels in the sPD cohorts since recent studies have indicated higher LRRK2 kinase activity^13^ and reduced GCase activity in sporadic PD^14^. Unlike the genetic cohorts studied here, the sPD group did not differ in baseline BMP levels from HCs. Thus, unfortunately, urinary BMP assessment would not inform the enrichment of sPD patients with endolysosomal deficits for therapeutic development. Although disappointing, this observation is consistent with our unbiased proteomic studies on urine^30^ and cerebrospinal fluid^31^ where we observed an enrichment in alterations in lysosome-associated proteins in sPD compared to HC in the cerebrospinal fluid but not the urine, despite seeing significant changes in both biometrics in LRRK2 G2019S+ individuals^30,31^. We also evaluated whether baseline BMP would prognosticate PD progression assessed by MDS-UPDRS III Off, MoCA, or striatal DaT SBR in either the genetic cohorts or the sPD cohort. We did not see an association between any BMP isoform and PD progression, but it is noteworthy that the *LRRK2* and *GBA1* genetic cohorts in PPMI show the minimal progression on these clinical and radiometric outcomes over 5 years since enrollment.

The source(s) of urinary BMP in the urine is an area of active research although recently direct secretion from kidneys has been suggested^30^. However, urinary BMP has been studied as a target modulation biomarker of LRRK2 kinase inhibitors in preclinical^32,33^ and clinical studies^29^. Here we demonstrate that in the *LRRK2* G2019S carriers, BMP isoforms remain stable over two years, strengthening its utility as a pharmacodynamic biomarker. To our knowledge, alterations in BMP isoforms have not been examined as a biomarker of GCase-targeted therapies, either preclinically or clinically. It would be interesting to study whether GCase activation can restore BMP levels in *GBA1* mutation carriers or in cellular and animal models of GCase deficiency. Clinical development of such therapeutics would be facilitated by biomarkers that enable the assessment of pharmacodynamic effects and patient enrichment.

In conclusion, our study shows that urinary BMP is a trait marker of *LRRK2* G2019S, *LRRK2* R1441G, and *GBA1* N409S variants with more robust elevations in BMP concentrations in *LRRK2* than *GBA1* carriers. The relative stability of BMP over two years in G2019S+ individuals strengthens its reported utility as a target modulation biomarker. The data provided here could facilitate power calculations for the assessment of BMP as a pharmacodynamic biomarker for LRRK2 or GCase-targeted therapies. However, we were not able to see the prognostic utility of baseline BMP on PD progression monitored by MDS-UPDRS III Off, MoCA, or striatal DaT imaging in either genetic or sPD cohorts of PPMI, precluding its use for patient enrichment or monitoring disease progression.

## Methods

### Study design

PPMI is an ongoing international, multicenter, observational study initiated in June 2010 with longitudinal follow-up as described previously^34,35^. The study was approved by the institutional review board at each site, and participants provided written informed consent. The primary aim of PPMI is to identify genomic, biochemical, or imaging biomarkers of clinical progression. PPMI data are publicly available (www.ppmi-info.org/data) and updated in real-time. The detailed study protocol, manuals, urine collection, and storage processes are available at www.ppmi-info.org/study-design.

### Participants and biospecimens included in the analyses

Urine aliquots from four cohorts classified by the presence/absence of pathogenic gene variants were analyzed: healthy controls (HC) and sPD without pathogenic variants, *LRRK2* mutation carriers with G2019S (G2019S+) or R1441G (R1441G+) manifesting PD and NMC and *GBA1* N409S carriers (N409S+) with PD and NMC. We focused the majority of statistical modeling on *LRRK2* (G2019S+, R1441G+) and *GBA1* N409S+ carriers. Note that for the purpose of these analyses, *GBA1* E365K and T408M were not considered pathogenic and a few individuals with one of these variants were present in G2019S+, N409S+, sPD, or HC cohorts analyzed here (see Supplementary Table 6). For *GBA1* variants other than N409S, we generated Descriptive Statistics only because of relatively small sample sizes. The additional *GBA1* variants analyzed are the pathogenic variant, L483P, a group of rare pathogenic variants combined together due to a small sample size (IVS2 + 1 G > A, L29Afs*18, T408M + R159W, and R502C) and the common risk variant, E365K. For the Descriptive Statistics, we have provided the specific variant information by disease status in the respective tables. Only those subjects who had baseline BMP data and non-missing genetic information were included in the analyses. In addition, the *LRRK2*+ cohort was analyzed for longitudinal changes (baseline, year 1 and year 2) in BMP levels.

### Study outcomes

All participants enrolled in PPMI undergo a standard test battery of clinical assessments described in detail previously^7,8^. In the current analyses, we focused on motor and cognitive outcomes using the Movement Disorders Society-Unified Parkinson’s Disease Rating Scale Part III OFF (MDS-UPDRS III Off) and Montreal Cognitive Assessment (MoCA) data up through year 5. In addition, we used dopamine transporter (DaT) SPECT imaging data (DaTscan) collected at baseline and then every other year. Specifically, mean striatal specific binding ratios (SBR) computed as described previously^33^ were incorporated into the analyses.

### Quantitative assessment of BMP isoforms in the urine

BMPs can exist in three geometrical isoforms (2, 2′-, 2, 3′-, and 3, 3′- BMP), which may influence their functional properties^36,37^. Based on our previous observation that among all the isoforms of BMP tested, di-18:1-BMP and total di-22:6-BMP most strongly discriminated the *LRRK2* mutation carriers from non-carriers^20^, we focused the current analyses on total di-18:1-BMP, total di-22:6-BMP (total = the sum of three isoforms) and 2, 2′- di-22:6-BMP. Measurements of BMP levels were performed by Nextcea, Inc. (Woburn, MA) using targeted UPLC-MS/MS and multiple reaction monitoring^26^ as described below. Urinary BMPs were extracted by liquid-liquid extraction using a SCIEX TripleTOF 6600 mass spectrometer equipped with an IonDrive Turbo V source (SCIEXm Framingham, MA). Injections were made using a Shimadzu Nexera XR UPLC system (Shimadzu Scientific Instruments, Japan). The instruments were controlled by AnalystTF 1.7 software. Quantitation was performed using authentic di-22:6-BMP and di-18:1-BMP reference standards. Internal standards were used for each analyte reported. The intensities of the analytes and internal standards were determined by the integration of extracted ion peak areas using AnalystTF 1.7 and MultiQuant 3.0 software. Calibration curves were prepared by plotting the peak area ratios for each analyte to internal standard versus concentration. The model for the calibration curve was linear with (1/x^2^) weighting. Each urine aliquot was assayed also for creatinine concentration by a colorimetric assay (method of Jaffé) with Parameter Creatinine Assay test reagents (R&D Systems, Minneapolis, MN) using a BioTek ELx800 absorbance microplate reader with Gen5 Microplate Reader and Imager Software 2.09 (Fisher Scientific, Hampton, NH)^20,26^. Concentrations of urine BMPs (ng/mL) were normalized to the concentration of urine creatinine and reported as ng/mg creatinine.

### Statistical analyses

Descriptive statistics of demographic and baseline characteristics were computed for each genetic variant group by disease status. For quantitative Gaussian characteristics, the means for each variant and disease status were modeled using Welch’s one-way analysis of variance (ANOVA). The overall *F*-test was examined for significance. If a significant difference was present, pairwise differences of interest were examined using Student’s *t*-test, assuming unequal variances. For quantitative non-Gaussian characteristics (except BMP), the Kruskal–Wallis test was used to examine an overall difference among groups. If a difference was present, pairwise differences of interest were examined using a Mann–Whitney *U*-test. Baseline BMP levels were compared using a rank-based linear model with adjustment for age and sex using a similar protected approach. For qualitative variables, a chi-square test was used to analyze proportional differences among the cohorts. A Bonferroni correction was applied to all pairwise comparisons for a specific characteristic as indicated in the result tables.

Longitudinal urine BMP levels between PD and NMC mutation carriers among the *LRRK2*+ cohort were compared using linear mixed models (LMMs). The log of the BMP level was modeled due to the skewed distribution. We included fixed effects of disease status (PD vs NMC), time (months), and interaction of disease status and time with adjustment for age and sex. We report the estimated annualized change in log BMP for PD and NMC as well as the difference between PD and NMC groups.

To assess the effect of baseline BMP measures on the longitudinal change in MoCA and MDS-UPDRS III Off within a specific variant group (G2019S+, R1441G+, or N409S+) or the sPD and HC combined group, we used longitudinal Tobit analysis. A separate model was fit for each group and included participants from both disease states. The models were fit using the SAS procedure Proc NLMixed with Adaptive Gaussian Quadrature (15 quadrature points). The parameter values from an LMM with a random intercept were used as starting values. For examining the effect of baseline BMP levels on mean striatal DaT SBR over time, we used LMMs. Fixed effects for time and baseline BMP, as well as the interaction of time and baseline BMP, were included in the longitudinal models for MoCA, MDS-UPDRS III Off, and mean striatal DaT SBR. All models adjusted for disease status, age, and sex. The MoCA models also adjusted for education. Akaike’s Information Criterion (AIC) was used to determine the inclusion of an interaction of disease status and time in each model. The main effect of interest was the interaction of time and baseline BMP.

Analyses use a significance level of 0.05 unless otherwise stated and all statistical tests were two-sided. Analyses were performed using SAS software Version 9.4 (SAS Institute, Cary, NC).

### Reporting summary

Further information on research design is available in the Nature Research Reporting Summary linked to this article.

## Supplementary information

Supplementary Tables

Reporting Summary

## Data Availability

PPMI is an open-access dataset. Data used in the preparation of this manuscript were obtained from the PPMI database (www.ppmi-info.org/data). Study protocol and manuals are available at www.ppmi-info.org/study-design. The data used for this paper were downloaded on Sept 3, 2021.
