# Supplementary material for: LRRK2 and GBA1 variant carriers have higher urinary bis(monacylglycerol) phosphate concentrations in PPMI cohorts
Source: NPJ Parkinsons Dis. 2023 Feb 28;9:30. doi: 10.1038/s41531-023-00468-2 (PMC9974978; doi:10.1038/s41531-023-00468-2)
Supplement: Supplementary file 1 — Supplementary Tables [file 41531_2023_468_MOESM1_ESM.docx]

**Supplementary Table 1. Comparison of baseline BMP Concentrations (adjusted for age and sex) among *LRRK2* G2019S+ and R1441G+ sub-cohorts by disease status**

|  | 1 | 2 | 3 | 4 |  |  |  |
| --- | --- | --- | --- | --- | --- | --- | --- |
|  | **G2019S+ PD**  **n = 134** | **G2019S+ NMC**  **n = 182** | **R1441G+ PD**  **n = 15** | **R1441G+ NMC**  **n = 15** | **P Value**  **all** | **P Value**  **1 vs 3** | **P Value**  **2 vs 4** |
| **Total di-18:1-BMP, median (range)** | 12.60 | 13.88 | 14.25 | 22.65 | 0.0343 | 0.3093 | **0.0092** |
|  | (1.29, 160.95) | (0.90, 71.97) | (5.04, 39.26) | (5.40, 72.51) |  |  |  |
| **Total di-22:6-BMP, median (range)** | 65.63 | 63.79 | 71.52 | 47.27 | 0.9078 |  |  |
|  | (9.85, 307.69) | (8.65, 245.60) | (30.90, 136.67) | (19.15, 121.85) |  |  |  |
| **2,2’-di-22:6-BMP, median (range)** | 51.39 | 49.38 | 49.43 | 36.29 | 0.8269 |  |  |
|  | (6.22, 240.68) | (6.99, 248.17) | (17.82, 116.30) | (11.50, 101.43) |  |  |  |

PD: Parkinson’s disease; NMC: non-manifesting carriers; BMP: bis(monoacylglycerol)phosphate

**Supplementary Table 2. Longitudinal BMP concentrations by disease status in *LRRK2* variant carriers**

| **Variant Group** | **BMP Isoform** | **Disease Status** | **Baseline** | **Year 1** | **Year 2** |
| --- | --- | --- | --- | --- | --- |
| ***LRKK2* G2019S+** | **Total di-18:1-BMP**  **median**  **(range)**  **Missing** | PD  n=134 | 12.60 | 13.14 | 15.29 |
|  |  |  | (1.29, 160.95) | (1.50, 64.80) | (1.39, 60.32) |
|  |  |  | 0 | 38 | 68 |
|  |  | NMC  n=182 | 13.88 | 13.02 | 14.83 |
|  |  |  | (0.90, 71.97) | (2.32, 99.02) | (1.65, 76.51) |
|  |  |  | 0 | 42 | 96 |
|  | **Total di-22:6-BMP**  **median**  **(range)**  **Missing** | PD  n=134 | 65.63 | 69.37 | 79.22 |
|  |  |  | (9.85, 307.69) | (12.49, 244.28) | (17.21, 298.52) |
|  |  |  | 0 | 38 | 68 |
|  |  | NMC  n=182 | 63.79 | 67.22 | 72.56 |
|  |  |  | (8.65, 245.60) | (7.45, 289.56) | (15.78, 320.75) |
|  |  |  | 0 | 42 | 96 |
|  | **2,2’-di-22:6-BMP median**  **(range)**  **Missing** | PD  n=134 | 51.39 | 50.09 | 64.62 |
|  |  |  | (6.22, 240.68) | (9.69, 205.83) | (8.75, 266.60) |
|  |  |  | 0 | 38 | 68 |
|  |  | NMC  n=182 | 49.38 | 46.95 | 57.07 |
|  |  |  | (6.99, 248.17) | (2.21, 244.24) | (12.75, 252.80) |
|  |  |  | 0 | 42 | 96 |
| ***LRRK2* R1441G+** | **Total di-18:1-BMP**  **median**  **(range)**  **Missing** | PD  n=15 | 14.25 | 17.61 | 18.31 |
|  |  |  | (5.04, 39.26) | (6.09, 36.67) | (10.48, 29.33) |
|  |  |  | 0 | 0 | 5 |
|  |  | NMC  n=15 | 22.65 | 17.40 | 26.46 |
|  |  |  | (5.40, 72.51) | (8.02, 70.62) | (5.47, 102.11) |
|  |  |  | 0 | 2 | 5 |
|  | **Total di-22:6-BMP**  **median**  **(range)**  **Missing** | PD  n=15 | 71.52 | 80.69 | 74.28 |
|  |  |  | (30.90, 136.67) | (25.13, 206.32) | (37.52, 196.85) |
|  |  |  | 0 | 0 | 5 |
|  |  | NMC  n=15 | 47.27 | 54.08 | 78.23 |
|  |  |  | (19.15, 121.85) | (24.04, 190.06) | (42.18, 153.86) |
|  |  |  | 0 | 2 | 5 |
|  | **2,2’-di-22:6-BMP median**  **(range)**  **Missing** | PD  n=15 | 49.43 | 65.35 | 56.47 |
|  |  |  | (17.82, 116.30) | (22.12, 159.15) | (26.03, 169.08) |
|  |  |  | 0 | 0 | 5 |
|  |  | NMC  n=15 | 36.29 | 39.25 | 57.36 |
|  |  |  | (11.50, 101.43) | (19.17, 139.44) | (28.22, 135.99) |
|  |  |  | 0 | 2 | 5 |

**Supplementary Table 3a. Baseline BMP concentrations (adjusted for age and sex) in rare* *GBA1*+ PD, rare *GBA1*+ NMC, sPD and HC**

|  | 1 | 2 | 3 | 4 |  |
| --- | --- | --- | --- | --- | --- |
|  | **Rare *GBA1*+ PD**  **n = 6** | **Rare *GBA1*+ NMC**  **n = 4** | **sPD**  **n = 379** | **HC**  **n = 190** | **P Value**  **all** |
| **Total di-18:1-BMP, median (range)** | 2.17 | 3.56 | 3.53 | 3.77 | 0.9457 |
|  | (0.97, 8.96) | (1.19, 13.44) | (0.21, 38.02) | (0.39, 22.03) |  |
| **Total di-22:6-BMP, median (range)** | 13.55 | 7.3 | 10.61 | 10.42 | 0.7525 |
|  | (1.27, 40.33) | (2.63, 22.03) | (1.18, 76.76) | (1.25, 85.89) |  |
| **2,2’-di-22:6-BMP, median (range)** | 9.98 | 4.25 | 6.83 | 6.37 | 0.7300 |
|  | (0.74, 27.92) | (1.78, 14.34) | (0.81, 59.27) | (0.60, 65.96) |  |

Rare variants include: IVS2+1G>A (N=2; 1 PD), L29Afs*18 (N=6; 3 PD): T408M+ R159W (N=1 PD) and R502C (N=1 PD)

**Supplementary Table 3b. Baseline BMP concentrations (adjusted for age and sex) in *GBA1* L483P+, sPD and HC**

|  | 1 | 2 | 3 | 4 |  |
| --- | --- | --- | --- | --- | --- |
|  | **L483P+PD***  **n = 8** | **L483P+NMC**  **n = 3** | **sPD**  **n = 379** | **HC**  **n = 190** | **P Value**  **all** |
| **Total di-18:1-BMP, median (range)** | 6.63 | 2.12 | 3.53 | 3.77 | 0.8667 |
|  | (0.84, 13.76) | (1.71, 8.52) | (0.21, 38.02) | (0.39, 22.03) |  |
| **Total di-22:6-BMP, median (range)** | 7.85 | 6.3 | 10.61 | 10.42 | 0.9133 |
|  | (4.05, 17.99) | (5.66, 35.28) | (1.18, 76.76) | (1.25, 85.89) |  |
| **2,2’-di-22:6-BMP, median (range)** | 4.96 | 4.18 | 6.83 | 6.37 | 0.5728 |
|  | (2.63, 9.23) | (3.76, 26.90) | (0.81, 59.27) | (0.60, 65.96) |  |

*Includes N=1 with E365K+ L483P+

**Supplementary Table 3c. Baseline BMP concentrations (adjusted for age and sex) in *GBA1* E365K+ compared to sPD and HC without E365K**

|  | 1 | 2 | 3 | 4 |  |
| --- | --- | --- | --- | --- | --- |
|  | **E365K+PD**  **n = 19** | **E365K+NMC**  **n = 5** | **E365K-sPD**  **n = 360** | **E365K-HC**  **n = 185** | **P Value**  **all** |
| **Total di-18:1-BMP, median (range)** | 4.19 | 1.07 | 3.53 | 3.78 | 0.8763 |
|  | (0.72, 20.40) | (0.78, 12.05) | (0.21, 38.02) | (0.39, 22.03) |  |
| **Total di-22:6-BMP, median (range)** | 8.81 | 8.80 | 10.71 | 10.47 | 0.7445 |
|  | (3.53, 22.67) | (7.51, 26.36) | (1.18, 76.76) | (1.25, 85.89) |  |
| **2,2’-di-22:6-BMP, median (range)** | 4.74 | 6.14 | 7.07 | 6.41 | 0.4778 |
|  | (2.52, 15.17) | (4.26, 11.31) | (0.81, 59.27) | (0.60, 65.96) |  |

PD: Parkinson’s disease; NMC: non-manifesting carriers; BMP: bis(monoacylglycerol)phosphate

**Supplementary Table 4. Baseline BMP concentrations (adjusted for age and sex) in (*LRRK2* G2019S and *GBA1* N409S)+ double variant carriers with PD or NMC compared to sPD and HC**

|  | 1 | 2 | 3 | 4 |  |  |  |
| --- | --- | --- | --- | --- | --- | --- | --- |
|  | **(G2019S, N409S)+ PD**  **n = 5** | **(G2019S, N409S)+ NMC***  **n = 15** | **sPD**  **n = 379** | **HC**  **n = 190** | **P Value**  **1 vs 2** | **P Value**  **1 vs 3** | **P Value**  **2 vs 4** |
| **Total di-18:1-BMP, median (range)** | 7.26 | 14.27 | 3.53 | 3.77 | 0.6412 | 0.1004 | **0.0095** |
|  | (3.16, 53.47) | (1.88, 50.10) | (0.21, 38.02) | (0.39, 22.03) |  |  |  |
| **Total di-22:6-BMP, median (range)** | 51.9 | 48.61 | 10.61 | 10.42 | 0.7910 | **<0.0001** | **<0.0001** |
|  | (28.22, 351.68) | (25.60, 328.84) | (1.18, 76.76) | (1.25, 85.89) |  |  |  |
| **2,2’-di-22:6-BMP, median (range)** | 44.62 | 37.14 | 6.83 | 6.37 | 0.4377 | **<0.0001** | **<0.0001** |
|  | (23.98, 228.44) | (5.00, 258.64) | (0.81, 59.27) | (0.60, 65.96) |  |  |  |

*****Includes one individual with G2019S+ N409S+/N409S+

Pairwise comparisons that showed significance at p value < 0.0125 (after Bonferroni correction)

are shown in bolded font.

PD: Parkinson’s disease; NMC: non-manifesting carriers; sPD: sporadic PD; HC: healthy control; BMP: bis(monoacylglycerol)phosphate

**Supplementary Table 5a. Outcome summary statistics by disease status over time in *LRRK2* G2019S+ cohort**

| **Outcome** | **Disease Status** | **Baseline** | **Year 1** | **Year 2** | **Year 3** | **Year 4** | **Year 5** |
| --- | --- | --- | --- | --- | --- | --- | --- |
| **Mean Striatal DaT SBR**       Mean (SD) | PD | 1.29 (0.38) | 1.39 (0.28) | 1.10 (0.32) | 0.78 (N/A) | 1.02 (0.29) |  |
| Missing | N=134 | 15 | 125 | 60 | 133 | 91 | 134 |
| Mean (SD) | NMC | 2.52 (0.52) | 3.35 (N/A) | 2.46 (0.55) | 2.61 (0.02) | 2.34 (0.53) |  |
| Missing | N=182 | 9 | 181 | 61 | 180 | 149 | 182 |
| **MDS-UPDRS III Off**       Mean (SD) | PD | 22.46 (11.45) | 23.98 (10.56) | 27.37 (12.26) | 28.78 (13.41) | 29.27 (13.56) | 30.03 (13.29) |
| Missing | N=134 | 36 | 43 | 63 | 85 | 93 | 105 |
| Mean (SD) | NMC | 3.14 (4.11) | 3.29 (4.14) | 3.26 (4.96) | 3.84 (5.24) | 4.98 (4.43) | 4.71 (3.79) |
| Missing | N=182 | 0 | 18 | 45 | 97 | 139 | 154 |
| **MoCA**       Median  (range) | PD | 27.00  (13.00, 30.00) | 27.00  (9.00, 30.00) | 27.00  (12.00, 30.00) | 27.00  (19.00, 30.00) | 27.00  (12.00, 30.00) | 28.00  (17.00, 30.00) |
| Missing | N=134 | 2 | 14 | 41 | 53 | 74 | 95 |
| Median  (range) | NMC | 27.00  (18.00, 30.00) | 27.50  (16.00, 30.00) | 28.00  (20.00, 30.00) | 28.00  (20.00, 30.00) | 28.00  (20.00, 30.00) | 27.00  (19.00, 30.00) |
| Missing | N=182 | 1 | 14 | 44 | 97 | 137 | 155 |

PD: Parkinson’s disease; NMC: non-manifesting carriers; sPD: sporadic PD; HC: healthy control; SD: standard deviation; DaT: dopamine transporter; SBR: specific binding ratio, MDS-UPDRS: Movement Disorders Society-Unified Parkinson’s disease Rating Scale; MoCA: Montreal Cognitive Assessment; BMP: bis(monoacylglycerol)phosphate.

**Supplementary Table 5b. Outcome summary statistics by disease status over time in *GBA1* N409S+ Cohort**

| **Outcome** | **Disease Status** | **Baseline** | **Year 1** | **Year 2** | **Year 3** | **Year 4** | **Year 5** |
| --- | --- | --- | --- | --- | --- | --- | --- |
| **Mean Striatal DaT SBR**       Mean (SD) | PD | 1.28 (0.52) | 1.13 (0.30) | 1.04 (0.34) |  | 0.81 (0.27) |  |
| Missing | N=76 | 13 | 69 | 35 | 76 | 63 | 76 |
| Mean (SD) | NMC | 2.79 (0.57) |  | 2.67 (0.53) |  | 2.54 (0.33) |  |
| Missing | N=178 | 5 | 178 | 81 | 178 | 170 | 178 |
| **MDS-UPDRS III Off**       Mean (SD) | PD | 26.87 (11.11) | 28.70 (13.67) | 30.18 (13.66) | 29.67 (13.94) | 33.71 (9.93) | 30.75 (13.02) |
| Missing | N=76 | 15 | 22 | 32 | 52 | 59 | 72 |
| Mean (SD) | NMC | 2.60 (3.90) | 2.50 (3.24) | 3.00 (4.21) | 2.75 (3.61) | 2.92 (2.99) | 3.00 (N/A) |
| Missing | N=178 | 1 | 20 | 68 | 127 | 165 | 177 |
| **MoCA**       Median  (range) | PD | 27.00  (15.00, 30.00) | 26.00  (14.00, 30.00) | 27.00  (21.00, 30.00) | 27.00  (18.00, 30.00) | 28.00  (18.00, 30.00) | 28.50  (23.00, 30.00) |
| Missing | N=76 | 0 | 5 | 24 | 42 | 58 | 70 |
| Median  (range) | NMC | 27.00  (16.00, 30.00) | 27.00  (20.00, 30.00) | 27.00  (16.00, 30.00) | 27.00  (21.00, 30.00) | 28.00  (25.00, 30.00) | 30.00  (30.00, 30.00) |
| Missing | N=178 | 0 | 16 | 64 | 125 | 163 | 177 |

PD: Parkinson’s disease; NMC: non-manifesting carriers; sPD: sporadic PD; HC: healthy control; SD: standard deviation; DaT: dopamine transporter; SBR: specific binding ratio, MDS-UPDRS: Movement Disorders Society-Unified Parkinson’s disease Rating Scale; MoCA: Montreal Cognitive Assessment; BMP: bis(monoacylglycerol)phosphate.

**Supplementary Table 5c. Outcome summary statistics by disease status over time in sPD and HC**

| **Outcome** | **Disease Status** | **Baseline** | **Year 1** | **Year 2** | **Year 3** | **Year 4** | **Year 5** |
| --- | --- | --- | --- | --- | --- | --- | --- |
| **Mean Striatal DaT SBR**       Mean (SD) | sPD | 1.39 (0.39) | 1.23 (0.35) | 1.15 (0.38) | 0.96 (0.27) | 1.02 (0.35) | 0.91 (0.47) |
| Missing | N=379 | 4 | 44 | 65 | 369 | 115 | 371 |
| Mean (SD) | HC | 2.57 (0.56) | 2.62 (0.08) |  |  | 2.13 (N/A) |  |
| Missing | N=190 | 3 | 188 | 190 | 190 | 189 | 190 |
| **MDS-UPDRS III Off**       Mean (SD) | sPD | 20.89 (8.79) | 25.12 (10.79) | 27.45 (11.12) | 29.41 (11.99) | 30.92 (12.20) | 31.54 (12.76) |
| Missing | N=379 | 0 | 82 | 122 | 142 | 143 | 170 |
| Mean (SD) | HC | 1.19 (2.15) | 1.62 (2.83) | 1.47 (2.78) | 1.45 (2.90) | 1.69 (3.22) | 2.54 (4.04) |
| Missing | N=190 | 2 | 11 | 22 | 29 | 33 | 39 |
| **MoCA**       Median  (range) | sPD | 28.00  (17.00, 30.00) | 27.00  (16.00, 30.00) | 27.00  (9.00, 30.00) | 27.00  (13.00, 30.00) | 27.00  (11.00, 30.00) | 27.00  (15.00, 30.00) |
| Missing | N=379 | 0 | 28 | 41 | 52 | 74 | 98 |
| Median  (range) | HC | 28.00  (26.00, 30.00) | 28.00  (20.00, 30.00) | 28.00  (21.00, 30.00) | 28.00 (19.00, 30.00) | 28.00  (19.00, 30.00) | 28.00  (20.00, 30.00) |
| Missing | N=190 | 0 | 11 | 22 | 28 | 33 | 39 |

**Supplementary Table 6. Pathogenic and non-pathogenic variants included in the main analyses by disease status**

| **Variant Groups for Main Analyses** | | | | | | | | |
| --- | --- | --- | --- | --- | --- | --- | --- | --- |
|  | ***LRRK2* G2019S+ (N=316)** | | ***LRRK2* R1441G+**  **(N=30)** | | **GBA1 N409S+**  **(N=254)** | | **No Pathogenic variants (N=569)** | |
| N by disease status | **N*=*134 PD** | **N*=*182 NMC** | **N=15 PD** | **N=15 NMC** | **N=76 PD** | **N=178 NMC** | **N=379 sPD** | **N=190 HC** |
| *N*: by variant | *132*: G2019S | *176*: G2019S | *15*: R1441G+ | *15*: R1441G | *69*: N409S+ | *164*: N409S+ | *350* | *182* |
| *N*: by variant | *2*: G2019S+ E365K+ | *4*: G2019S+ E365K+ |  |  | *1*: E365K+ N409S+ | *1*: E365K+ N409S+ | *19*: E365K+ | *5*: E365K+ |
| *N*: by variant |  | *2*: G2019S+ T408M+ |  |  | 6: N409S/N409S+ | *13*: N409S/N409S+ | *10*: T408M+ | *3*: T408M+ |
