# Supplementary material for: LRRK2 and GBA1 variant carriers have higher urinary bis(monacylglycerol) phosphate concentrations in PPMI cohorts
Source: NPJ Parkinsons Dis. 2023 Feb 28;9:30. doi: 10.1038/s41531-023-00468-2 (PMC9974978; doi:10.1038/s41531-023-00468-2)
Supplement: Supplementary file 2 — Reporting Summary [file 41531_2023_468_MOESM2_ESM.pdf]

nature portfolio

Corresponding author(s): Kalpana M. Merchant*, Tanya Simuni, Janel Fedler, Chelsea Caspell-Garcia, Michael Brumm, Kelly Nudelman, Elizabeth Tengstrandt, Frank Hsieh, Roy N. Alcalay, Christopher Coffey, Lana Chahine, Tatiana Foroud, Andrew Singleton, Daniel Weintraub, Samantha Hutten, Todd Sherer, Andrew Siderowf, Caroline Tanner, and Ken Marek on behalf of the PPMI Investigators

Last updated by author(s): 1/14/2023

Reporting Summary

Nature Portfolio wishes to improve the reproducibility of the work that we publish. This form provides structure for consistency and transparency in reporting. For further information on Nature Portfolio policies, see our Editorial Policies and the Editorial Policy Checklist.

Statistics

For all statistical analyses, confirm that the following items are present in the figure legend, table legend, main text, or Methods section.

n/a	Confirmed	
☐	☒	The exact sample size (*n*) for each experimental group/condition, given as a discrete number and unit of measurement
☐	☒	A statement on whether measurements were taken from distinct samples or whether the same sample was measured repeatedly
☐	☒	The statistical test(s) used AND whether they are one- or two-sided<br>*Only common tests should be described solely by name; describe more complex techniques in the Methods section.*
☐	☒	A description of all covariates tested
☐	☒	A description of any assumptions or corrections, such as tests of normality and adjustment for multiple comparisons
☐	☒	A full description of the statistical parameters including central tendency (e.g. means) or other basic estimates (e.g. regression coefficient) AND variation (e.g. standard deviation) or associated estimates of uncertainty (e.g. confidence intervals)
☐	☒	For null hypothesis testing, the test statistic (e.g. *F*, *t*, *r*) with confidence intervals, effect sizes, degrees of freedom and *P* value noted<br>*Give P values as exact values whenever suitable.*
☒	☐	For Bayesian analysis, information on the choice of priors and Markov chain Monte Carlo settings
☒	☐	For hierarchical and complex designs, identification of the appropriate level for tests and full reporting of outcomes
☐	☒	Estimates of effect sizes (e.g. Cohen's *d*, Pearson's *r*), indicating how they were calculated

Our web collection on statistics for biologists contains articles on many of the points above.

Software and code

Policy information about availability of computer code

Data collection	No software/code was used to collect the data in this study
Data analysis	SAS version 9.4 (SAS/STAT 15.1; SAS Institute Inc., Cary, NC)

For manuscripts utilizing custom algorithms or software that are central to the research but not yet described in published literature, software must be made available to editors and reviewers. We strongly encourage code deposition in a community repository (e.g. GitHub). See the Nature Portfolio guidelines for submitting code & software for further information.

Data

Policy information about availability of data

All manuscripts must include a data availability statement. This statement should provide the following information, where applicable:

- Accession codes, unique identifiers, or web links for publicly available datasets
- A description of any restrictions on data availability
- For clinical datasets or third party data, please ensure that the statement adheres to our policy

PPMI is an open access dataset. Data used in the preparation of this manuscript were obtained from the PPMI database (www.ppmi-info.org/data). Study protocol and manuals are available at www.ppmi-info.org/study-design. The data used for this paper were downloaded September 3, 2021.

Human research participants

Policy information about studies involving human research participants and Sex and Gender in Research.

Reporting on sex and gender	Sex was determined based on self-reporting. The frequency of reported biological sex for each cohort is provided in Tables 1a, 1b, and 3. All models adjusted for sex.
Population characteristics	Population characteristics are presented in Tables 1a, 1b, and 3.
Recruitment	Participants were recruited by each site as described at www.ppmi-info.org/study-design.
Ethics oversight	The study was approved by the institutional review board at each site. The Institute for Neurodegenerative Disorders (IND; New Haven, CT) provides central regulatory oversight for PPMI.

Note that full information on the approval of the study protocol must also be provided in the manuscript.

Field-specific reporting

Please select the one below that is the best fit for your research. If you are not sure, read the appropriate sections before making your selection.

☒ Life sciences ☐ Behavioural & social sciences ☐ Ecological, evolutionary & environmental sciences

For a reference copy of the document with all sections, see nature.com/documents/nr-reporting-summary-flat.pdf

Life sciences study design

All studies must disclose on these points even when the disclosure is negative.

Sample size	No a priori sample size calculations were performed. Statistical modeling focused on LRRK2 G2019S+ (PD N=134; NMC N=182), LRRK2 R1441G + (PD N=15; NMC N=15), GBA1 N409S (PD N=76; NMC N=178), sPD (N=178) and HC (N=190) while only descriptive statistics were generated for E365K and other rare variants in GBA1 due to the limited data available.
Data exclusions	As described in the 'Participants and biospecimens included the analyses' paragraph of the Methods section, analyses excluded participants with missing baseline BMP data and missing genetic information. These exclusions were made as BMP and mutation were the focus of this analysis.
Replication	The analyses assessing the effects of LRRK2 pathogenic variant on baseline urinary BMP levels in PD manifesting and NMC were based on the previous observation of Alcalay et al (2020). All other analyses were not replicated, however, detailed methods are provided with the intent that further investigations of urinary BMP may be conducted.
Randomization	Allocation into experimental groups was not random. All modeling controlled for age and sex. As applicable models also adjusted for disease status and education.
Blinding	This observational, non-interventional study did not include blinding.

Reporting for specific materials, systems and methods

We require information from authors about some types of materials, experimental systems and methods used in many studies. Here, indicate whether each material, system or method listed is relevant to your study. If you are not sure if a list item applies to your research, read the appropriate section before selecting a response.

Materials & experimental systems

n/a	Involved in the study	
☒	☐	Antibodies
☒	☐	Eukaryotic cell lines
☒	☐	Palaeontology and archaeology
☒	☐	Animals and other organisms
☒	☐	Clinical data
☒	☐	Dual use research of concern

Methods

n/a	Involved in the study	
☒	☐	ChIP-seq
☒	☐	Flow cytometry
☒	☐	MRI-based neuroimaging
